# Nomenclatural review of names published in the fungal genus *Dermoloma* (Basidiomycota, Agaricales, Tricholomataceae) based on morphological analyses of type specimens

**DOI:** 10.3897/BDJ.13.e158080

**Published:** 2025-07-17

**Authors:** Michaela Caboňová, Marisol Sanchez-Garcia, Miroslav Caboň, Katarína Adamčíková, Pierre-Arthur Moreau, Alfredo Vizzini, Slavomír Adamčík, Soňa Jančovičová

**Affiliations:** 1 Department of Microbial and Plant Interactions, Plant Science and Biodiversity Centre, Slovak Academy of Sciences, Bratislava, Slovakia Department of Microbial and Plant Interactions, Plant Science and Biodiversity Centre, Slovak Academy of Sciences Bratislava Slovakia; 2 Department of Forest Mycology and Plant Pathology, Swedish University of Agricultural Sciences, Uppsala, Sweden Department of Forest Mycology and Plant Pathology, Swedish University of Agricultural Sciences Uppsala Sweden; 3 Department of Plant Pathology and Mycology, Institute of Forest Ecology, Slovak Academy of Sciences, Zvolen, Slovakia Department of Plant Pathology and Mycology, Institute of Forest Ecology, Slovak Academy of Sciences Zvolen Slovakia; 4 Laboratoire de Génie Civil et géo-Environnement, University of Lille, Lille, France Laboratoire de Génie Civil et géo-Environnement, University of Lille Lille France; 5 Department of Life Sciences and Systems Biology, University of Turin, Turin, Italy Department of Life Sciences and Systems Biology, University of Turin Turin Italy; 6 Department of Botany, Faculty of Natural Sciences, Comenius University Bratislava, Bratislava, Slovakia Department of Botany, Faculty of Natural Sciences, Comenius University Bratislava Bratislava Slovakia

**Keywords:** pileipellis, morphology, degraded DNA, fungaria, grassland agarics

## Abstract

**Background:**

The majority of members of the fungal genus *Dermoloma* have been described, based on morphology and without molecular support. Sequencing most *Dermoloma* type specimens has been unsuccessful, probably due to degraded DNA, leaving their taxonomy primarily reliant on morphological characters. In this study, we re-described nine *Dermoloma* types, providing standardised morphological descriptions that include observations of previously undocumented microscopic structures.

**New information:**

The pileipellis structure of *D.hybridum*, *D.inconspicuum* and D.intermediumvar.coniferarum differs strongly from the typical *Dermoloma* pileipellis and these taxa do not belong to this genus. *Dermolomaatrobrunneum* and *D.hymenocephalum* are distinct taxa which have not been reported recently. The concept of Dermolomacuneifoliumvar.punctipes is based on the presence of dark spots on the stipe. However, our examination of the type material reveals that its spores are more consistent with those of *D.atrocinereum*, a species that can also exhibit dark dots. *Dermolomalongibasidiatum* is likely a synonym of this species as well. The name *D.pseudocuneifolium* has been misapplied for a species with amyloid spores, but the type has inamyloid narrow spores characteristic for *D.bellerianum*. *Dermolomapragense* probably represents a distinct, but recently unrecorded European species defined by large basidiomata and small spores. The data presented here are essential for future nomenclatural treatments within *Dermoloma*, as current phylogenetic studies suggest the presence of a large number of undescribed species.

## Introduction

*Dermoloma* (J.E. Lange) Singer ex Herink (Basidiomycota, Tricholomataceae) is a genus of agaric fungi defined by small- to medium-sized basidiomata with a collybioid to tricholomatoid habit, dull grey and brownish colouration on pileus and stipe, lamellae generally emarginate and adnate in their attachment to stipes and the context with a distinct farinaceous odour. Microscopically, the genus is characterised by a pluristratous (multi-layered) hymenidermic pileipellis and the presence of clamp connections on hyphal septa (Sánchez-García et al. 2021). *Dermoloma* members are soil fungi growing in different types of grassland and forest ecosystems. The genus is included in the indicator group of so-called CHEGD fungi (acronym of taxon names Clavariaceae, *Hygrocybe* s.l., Entolomataceae, Geoglossaceae and *Dermoloma*) which are biotrophic fungi probably forming an unspecified symbiosis with plants (Caboň et al. 2021).

The previous complex taxonomic study of *Dermoloma* members, based strictly on morphology, included eight species in Europe (Contu et al. 2008). Sánchez-García et al. (2021) published first molecular phylogeny of the genus which included 25 European and six North American species-rank clades Voto (2022). The recent monographic study of the genus (Adamčíková et al. 2025) described 30 species from Europe (including 16 new species) and three from North America (all of them new to science) supported by molecular analysis of six DNA regions. No such comprehensive morphological study exists for *Dermoloma* species outside Europe.

Amongst 28 valid *Dermoloma* names at species and lower rank, 22 were published from Europe and six from the Americas (Sánchez-García et al. 2021). In that study, authors sequenced 19 *Dermoloma* types from Europe and North America, of which 10 attempts were successful. These 10 types correspond to seven *Dermoloma* species, because two names are synonyms and one is referring to a fungus from a different genus (Adamčíková et al. 2025). This study presents descriptions of nine *Dermoloma* types not described in Adamčíková et al. (2025) providing standardised and detailed micromorphological data with comments about their classification and distinguishing characters. These types were either not successfully sequenced or their sequences did not match recently collected material from Europe or North America. Older names with insufficient original diagnoses and sometimes with changing taxonomic concepts are actually hampering progress of new species descriptions because the process of sorting the taxonomic identity of these names requires time-consuming loan communication and taxonomic expertise (Dayarathne et al. 2016). However, overlooking old names when publishing new species due to relying strictly on sequence data may result in negative consequences (Koukol and Delgado 2021).

## Materials and methods

### Sampling and morphological study

The types of nine *Dermoloma* taxa were loaned and studied (Table 1). Amongst these types, only *D.hymenocephalum* and D.intermediumvar.coniferarum were successfully sequenced in a previous study by Sánchez-García et al. (2021).

Microscopic structures were examined in a solution of ammoniacal Congo red after a short treatment in aqueous 10% potassium hydroxide (KOH). The amyloid reaction of spore walls was assessed in Melzer’s reagent and observed using both the standard approach with initial observation immediately after mounting the object in the reagent (Melzer 1924) and also following a prolonged (30 min) incubation (with pre-heating) in Melzer’s reagent, as described by Vizzini et al. (2020). Pileipellis elements near the pileus margin and the pileus centre were observed and measured separately.

All microscopic observations followed standards of Adamčíková et al. (2025) and were observed under an Olympus CX-41 microscope with an oil-immersion lens at a magnification of 1000×. All drawings of microscopic structures, except for spores, were made with a camera lucida using an Olympus U-DA drawing attachment at a projection scale of 2000×. Spores on lamella sides not attached to basidia were observed and illustrated using QuickPHOTO MICRO 3.2 software visualising images captured by a Promicra 3-3CP camera. Enlarged scanned pictures of spores were used for measuring with an accuracy of 0.1 μm and for preparation of line drawings. Terminology of spore shapes follows Vellinga (1988). All other elements were measured with an accuracy of 0.5 μm. Spores, terminal elements in the pileipellis and caulocystidia were measured 30 times per collection, while basidia and marginal cells at lamellae edges were measured 20 times. The microscopic dimensions in the description are presented as a mean value plus/minus standard deviation, with minimum or maximum values. Q is the length/width ratio of spores.

### Molecular analysis of type specimens

We attempted to sequence ITS region or its fragments (ITS1/ITS2) for all analysed specimens. Molecular workflow (DNA extraction, PCR amplification, Sanger sequencing) followed Adamčíková et al. (2025). In case of succesful sequencing of ITS region, the respective sequence was deposited in GenBank (https://www.ncbi.nlm.nih.gov/genbank/) and accession number is indicated after reference in the Nomenclature section.

## Taxon treatments

### Dermoloma atrobrunneum

(Dennis) Singer ex Bon, 1986

F649E491-371B-5EC1-BD4D-DD8E9319524C

Dermoloma atrobrunneum (Dennis) Singer ex Bon, Doc. Mycol. 65: 51. 1986.
Basionym: *Tricholomaatrobrunneum* Dennis, Trans. Brit. Mycol. Soc. 34: 476. 1951.

#### Materials

- **Type status:** Holotype. **Occurrence:** occurrenceID: D07725D6-541C-545B-8535-560119D33AAB; **Location:** country: Trinidad; municipality: St. Joseph; locationRemarks: solitary on the ground in bamboo plantation; **Identification:** identifiedBy: R. W. G. Dennis; dateIdentified: 29-09-1949; **Record Level:** collectionID: *Dennis 8* [K(M) 37579]; institutionCode: K (M)

#### Description

**Figure 1.**
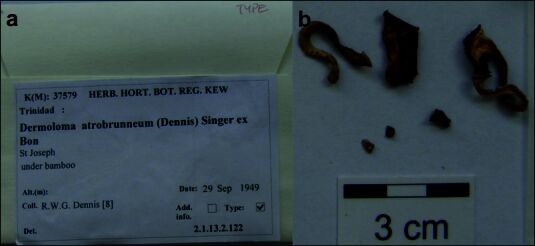
*Dermolomaatrobrunneum* [K(M) 37579, holotype], details of the type specimen; **a**: Original label of the type specimen; **b**: Basidiomata of the type collection.

**Figure 2.**
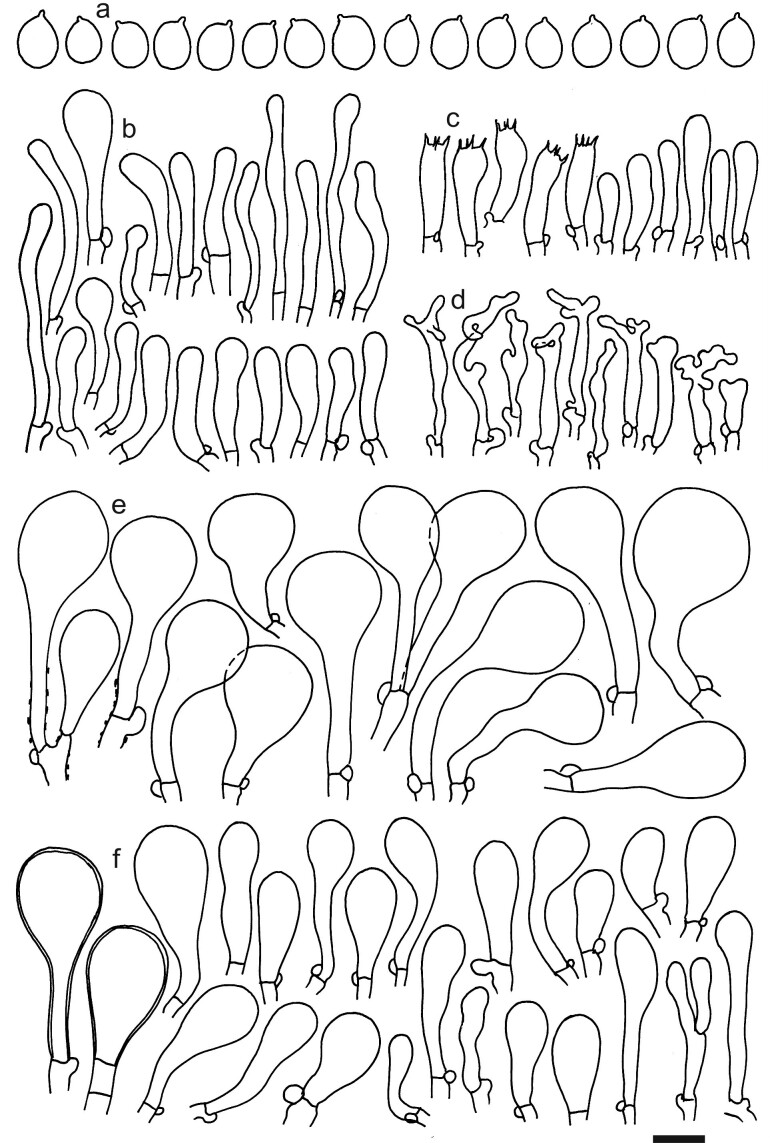
*Dermolomaatrobrunneum* [K(M) 37579, holotype], microscopic elements. Scale bar = 5 µm for spores and 10 µm for the other elements. **a** Spores; **b** Caulocystidia; **c** Basidia and basidioles; **d** Marginal cells; **e** Pileipellis elements near pileus margin; **f** Pileipellis elements near pileus centre.

Spores (4.7–)4.9–5.0–5.2(–5.3) × (3.6–)3.8–4.0–4.2(–4.4) μm; broadly ellipsoid, Q = (1.16–)1.21–1.26–1.31(–1.37); walls amyloid, thin-walled; hilar appendage ca. 0.5 μm long. Basidia (16–)18.5–20.6–23(–24) × (4.5–)5.5–6.2–7 μm; clavate; with 4 sterigmata. Basidioles first cylindrical, then clavate, ca. 4–6 μm wide. Marginal cells (12–)20–24.7–30(–34) × (2.5–)3–3.4–4(–5) μm; cylindrical or sometimes fusiform, apically diverticulate, branched or coralloid. Pileipellis of mainly one, occasionally two layers of inflated cells; hyphal terminations with brownish parietal pigments, occasionally with thickened walls up to 1 μm, near septa sometimes with incrusted dark brown pigments. Terminal cells near pileus margin (27–)30–37.8–45.5(–55) × (12.5–)14.5–17.1–19.5(–24.5) μm; usually sphaeropedunculate, sometimes obpyriform; subterminal cells usually narrower and implemented in intricate hyphae of subpellis. Terminal cells near pileus centre (15–)19–27.2–35(–46) × (2.5–)5.5–9–12.5(–18.5) μm; usually clavate, sometimes obpyriform, rarely sphaeropedunculate. Caulocystidia (15–)18.5–28.6–38.5(–60) × (3–)3.5–5.2–6.5(–10) μm; clavate, rarely cylindrical, slightly flexuous, apically obtuse, clustered in small ascending fascicules, sometimes individual and repent; usually thin-walled. Clamp connections present (Figs 1, 2).

#### Notes

*Dermolomaatrobrunneum* was described from Trinidad (Dennis 1951). Sequencing of the type specimen failed, but its morphology clearly confirmed the presence of amyloid spores and a cellular pileipellis typical for D.subgen.Amylospora Adamčík. It is the type species of D.sect.Atrobrunnea Contu, which is classified in this subgenus. The species differs clearly from the other amyloid-spored North American species in having small, broadly ellipsoid spores, measuring on average 5.0 × 4.0 μm, Q = 1.26 and in diverticulate, branched or coralloid, narrow (ca. 3–4 μm wide) marginal cells.

### Dermoloma cuneifolium cuneifolium

Arnolds 1992

FA70C4E1-6DF5-5FF8-A3B7-CEF78AE0EFD0

Dermoloma cuneifolium var. punctipes Arnolds, Persoonia 14(4): 529. 1992.

#### Materials

- **Type status:** Holotype. **Occurrence:** occurrenceID: 722E4591-6CF3-524B-941B-26C6E8EBC82D; **Location:** country: The Netherlands; stateProvince: Limburg; municipality: Wijlre, “Wrakelberg”; locationRemarks: in poorly developing hayfield on former arable land on steep calcareous slope with SW exposure; **Identification:** identifiedBy: E. Arnolds; dateIdentified: 22.10.1984; **Record Level:** collectionID: Arnolds 5337 (L0821553); institutionCode: L

#### Description

**Figure 3.**
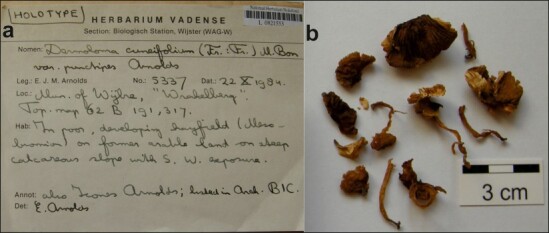
Dermolomacuneifoliumvar.punctipes (L0821553, holotype), details of the type specimen. **a** Original label of the type specimen; **b** Basidiomata of the type collection.

**Figure 4.**
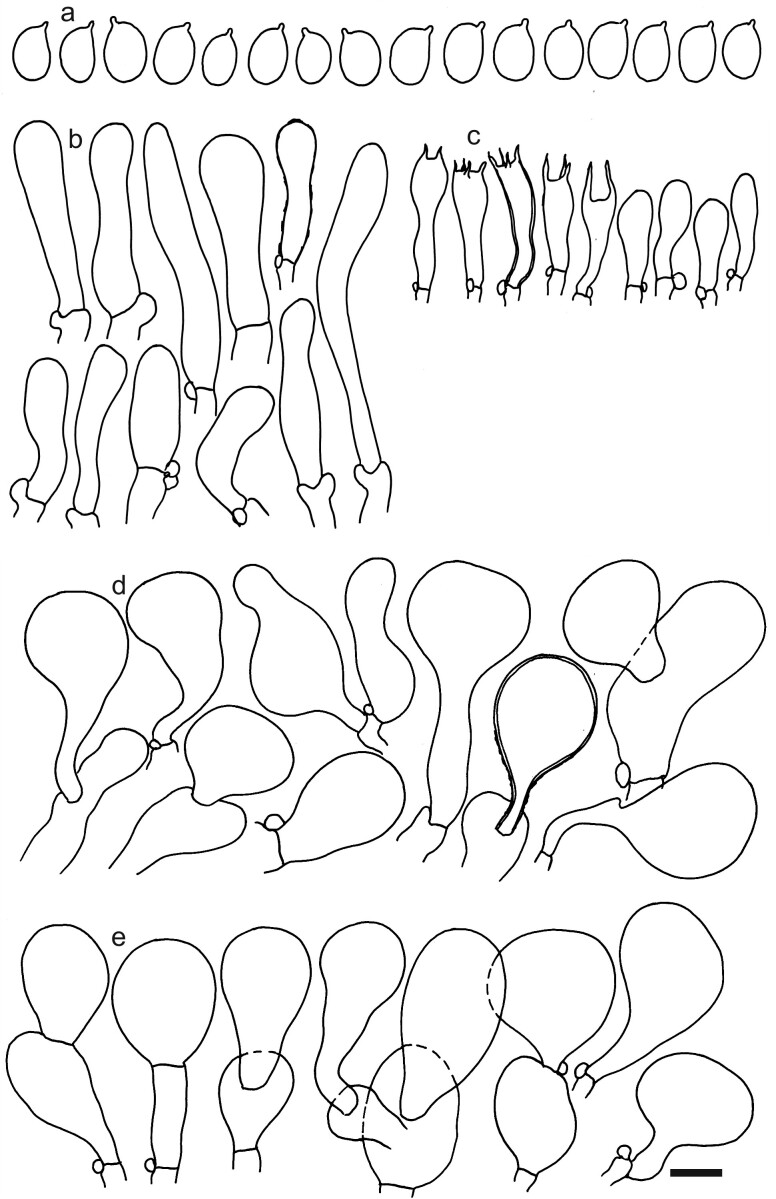
Dermolomacuneifoliumvar.punctipes (L0821553, holotype), microscopic elements. Scale bar = 5 µm for spores and 10 µm for the other elements. **a** Spores; **b** Caulocystidia; **c** Basidia and basidioles; **d** Pileipellis elements near the pileus margin; **e** Pileipellis elements near the pileus centre.

Spores (5.4–)5.6–6.0–6.4(–7.1) × (4.1–)4.2–4.4–4.6(–4.8) μm; broadly ellipsoid to ellipsoid, Q = (1.27–)1.29–1.37–1.44(–1.55); dextrinoid, inamyloid, thick-walled (walls ca. 0.5 μm); hilar appendage ca. 0.6–0.8 μm long. Basidia (7.5–)15–19.6–24(–27) × (4.5–)5–6.0–7(–7.5) μm; clavate, often thick-walled and dextrinoid; mostly with 4, occasionally with 2 and rarely with 3 sterigmata. Basidioles first cylindrical, then clavate, ca. 5–7 μm wide. Marginal cells not differentiated, similar to basidioles on lamellae sides, mixed with dispersed basidia. Pileipellis of mainly one, occasionally two layers of inflated cells; hyphal terminations with brownish parietal pigments, occasionally with thickened walls up to 1 μm, near septa often with incrusted dark brown pigments. Terminal cells near pileus margin (17–)25.5–37.2–48.5(–61) × (11–)16.5–20.8–25(–28) μm; sphaeropedunculate, obpyriform or utriform, often lobate; subterminal cells often lobate, usually narrower and implemented in intricate hyphae of subpellis. Terminal cells near pileus centre (14.5–)20–28.5–36.5(–43) × (12.5–)15.5–18.9–22.5(–26.5) μm; usually obpyriform, subglobose or ellipsoid, occasionally sphaeropedunculate. Caulocystidia (22–)25.5–38.1–50.5(–73) × (5.5–)7.3–9.2–11(–13.5) μm; clavate or cylindrical, sometimes centrally constricted, apically obtuse, repent with ascending tips and often clustered; usually thin-walled, often thick-walled and with incrusted dark brown pigments. Clamp connections present (Figs 3, 4).

#### Notes

Dermolomacuneifoliumvar.punctipes was originally recognised from the type variety by darker punctuations of the stipe and darker incrusted pigments on the caulocystidia (Arnolds 1992). We did not observe these characters in any collections identified as *D.cuneifolium* from sequence data; thus, D.var.punctipes probably corresponds to another *Dermoloma* species. Stipes with darker granulations were observed in several species with inamyloid spores, for example, *D.atrocinereum*, but these darker dots were usually near the stipe base. Sequencing of the type of D.var.punctipes failed and sequences of recent collections with darker spots on stipes, identified as D.var.punctipes, resulted in matches with at least three different species. Spores of the type specimen are on average 6 × 4.4 μm large, which is the best match for *D.atrocinereum* amongst species with dark punctuations on the stipe. We did not decide about the taxonomic status of this variety, but it is clear that the taxonomic concept, based only on the presence of darker dots on the stipe, corresponds to more than one species.

### Dermoloma hybridum

(Kühner) Bon, 1979

ABBF87DA-B99A-5451-9585-13D7548AC11F

Dermoloma hybridum (Kühner) Bon, Bulletin Annuel de la Fédération Centre-Est d'Histoire Naturelle et de Mycologie 1: 14. 1979.
Basionym: *Tricholomahybridum* Kühner, Ann. Sci. Franche-Comté 2: 31. 1947.

#### Materials

- **Type status:** Holotype. **Location:** country: France; stateProvince: Doubs; municipality: Besançon; locality: bois d'Avoudrey; locationRemarks: under fir mixed with deciduous trees; **Identification:** identifiedBy: R. Kühner; dateIdentified: 16-10-1946; **Record Level:** collectionID: G00126676; institutionCode: G

#### Description

**Figure 5.**
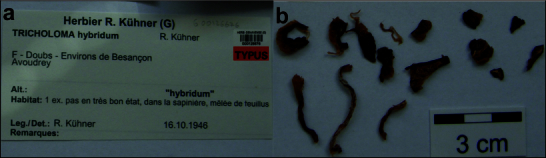
*Dermolomahybridum* (G00126676, holotype), details of the type specimen. **a** Original label of the type specimen; **b** Basidiomata of the type collection.

**Figure 6.**
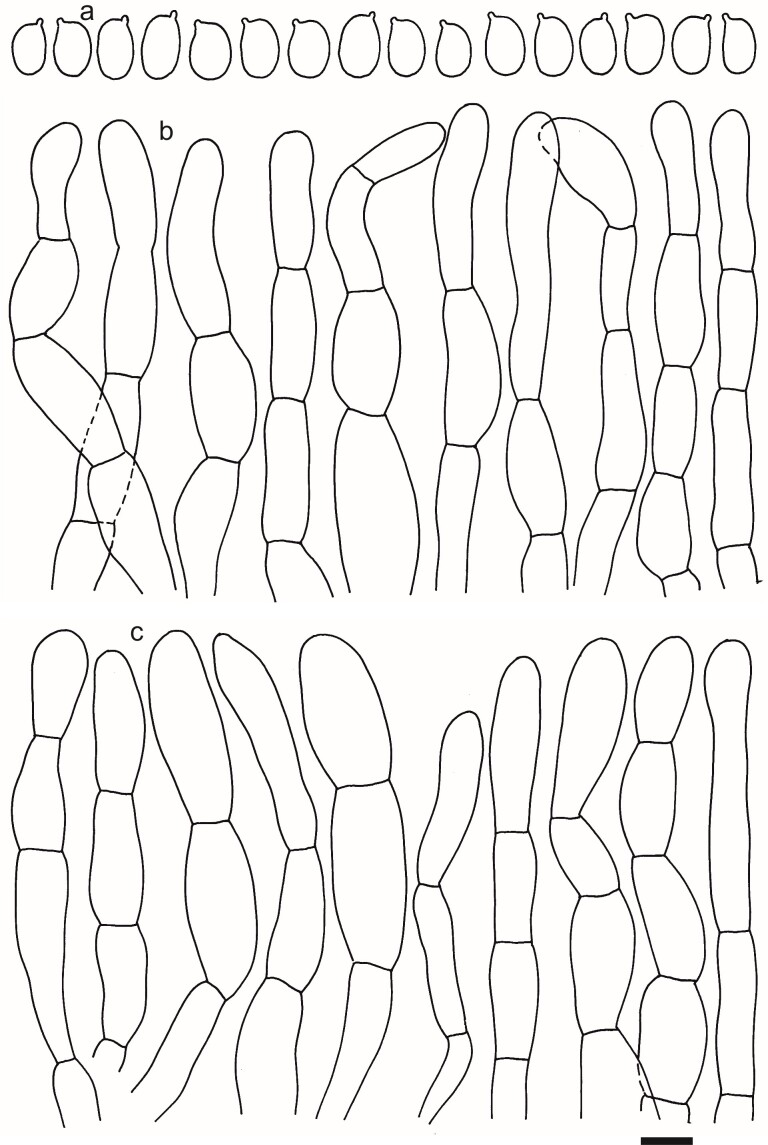
*Dermolomahybridum* (G00126676, holotype), microscopic elements. Scale bar = 5 µm for spores and 10 µm for the other elements. **a** Spores; **b** Pileipellis elements near the pileus margin; **c** Pileipellis elements near the pileus centre.

Spores (5.2–)5.4–5.8–6.2(–6.7) × (3.4–)3.6–3.9–4.2(–4.4) μm; ellipsoid to narrowly ellipsoid, Q = (1.36–)1.40–1.49–1.59(–1.73); inamyloid, not dextrinoid, thin-walled; hilar appendage ca. 0.5–1 μm long. Basidia and other elements of the hymenium not well preserved. Pileipellis a cutis of repent hyphae, composed of chains of inflated, ellipsoid, cylindrical or clavate cells; hyphal terminations thin-walled, sometimes with brownish granulose pigments. Terminal cells near pileus margin (20–)22–33.4–44(–62) × (7–)8–9.4–11(–13) μm; clavate, cylindrical or ellipsoid, apically obtuse; subterminal cells usually equal in size and shape. Terminal cells near pileus centre (20–)25.5–35.0–44.5(–62) × (8.5–)9.5–11.3–13.5(–17) μm; ellipsoid, obpyriform or cylindrical, rarely clavate. Caulocystidia not observed. Clamp connections not observed (Figs 5, 6).

#### Notes

*Dermolomahybridum* was described as *Tricholomahybridum* by Kühner (1947) and defined by a pileus 70–80 mm in diam., context with no odour and a suprapellis (referred in the original description as epicutis) of cylindrical hyphae. Bon (1979) combined this species in *Dermoloma*, although these characters clearly contradict the definition of the genus. Amplification of the barcode ITS region failed, but our study confirmed that the pileipellis structure is a cutis composed of chains of ellipsoid inflated cells, which is more typical for other *Tricholomataceae* members, including the genus *Tricholoma*.

### Dermoloma hymenocephalum

Singer, 1962

1E0CB9AF-168B-5F7C-ADB3-0FBAA5773929

Dermoloma hymenocephalum Singer, Sydowia 15(1-6): 142. 1962.
Basionym: *Collybiahymenocephala* A.H. Sm., Pap. Michigan Acad. Sci. 26: 61. 1941.
Synonyms: ≡ Replaced synonym: *Collybiahymenocephala* A.H. Sm., Pap. Michigan Acad. Sci. 26: 61. 1941, nom. Ileg.; non *Collybiahymenocephala* (Speg.) Speg., Syll. Fung. 5: 242 (1887).
≡ *Hydropushymenocephalus* (A.H. Sm.) Redhead, Sydowia 37: 266. 1984.
≡ *Mycenahymenocephala* (A.H. Sm.) A.H. Sm., North American Species of *Mycena*: 385. 1947.

#### Materials

- **Type status:** Holotype. **Location:** country: USA; county: near Dexter; municipality: Silver Lake; **Identification:** identifiedBy: A. H. Smith; dateIdentified: 23-09-1938; **Record Level:** collectionID: *Smith 11050* (MICH10228); institutionCode: MICH

#### Description

**Figure 7.**
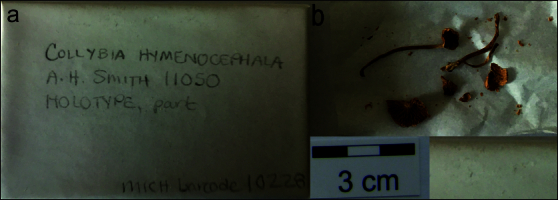
*Dermolomahymenocephalum* (MICH 10228, holotype), details of the type specimen. **a** Original label of the type specimen; **b** Basidiomata of the type collection.

**Figure 8.**
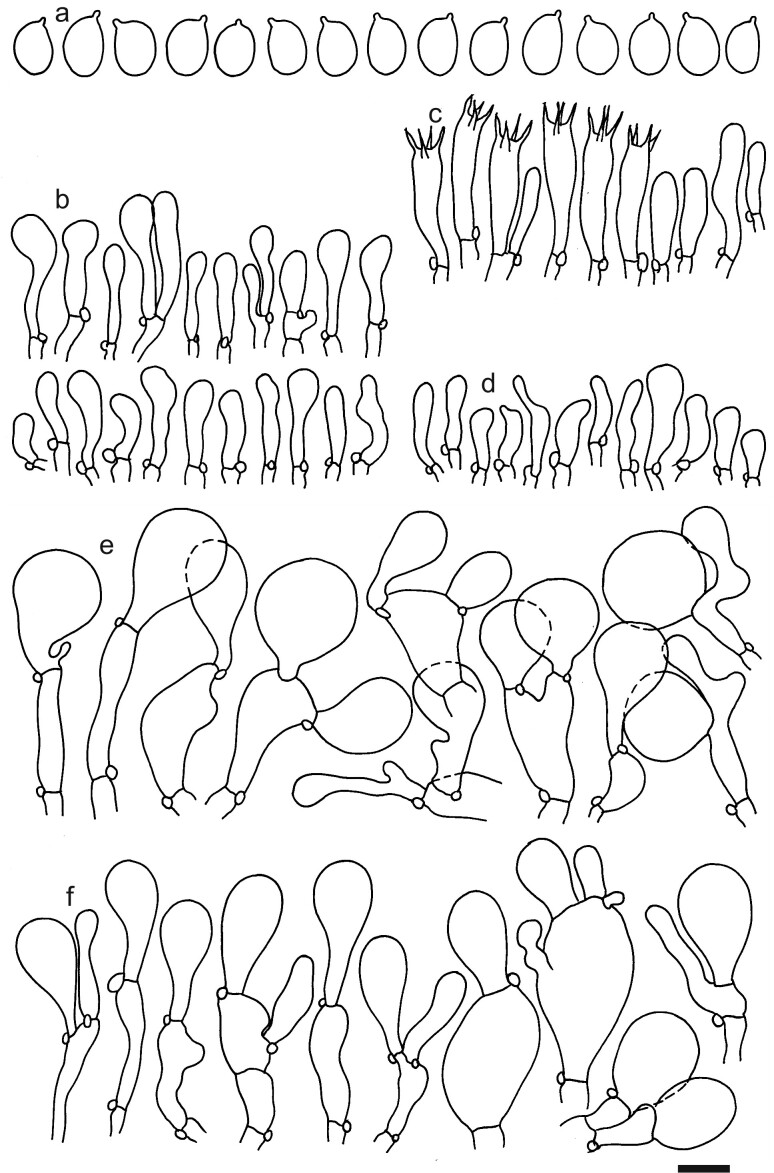
*Dermolomahymenocephalum* (MICH 10228, holotype), microscopic elements. Scale bar = 5 µm for spores and 10 µm for the other elements. **a** Spores; **b** Caulocystidia; **c** Basidia and basidioles; **d** Marginal cells; **e** Pileipellis elements near the pileus margin; **f** pileipellis elements near the pileus centre.

Spores (4.8–)5.4–5.9–6.4(–6.7) × (3.7–)3.9–4.2–4.5(–5.0) μm; ellipsoid to narrowly ellipsoid, Q = (1.24–)1.31–1.40–1.49(–1.61); amyloid, thin-walled; hilar appendage ca. 0.5–0.8 μm long. Basidia (24.5–)25.2–28.0–30(–34) × (5.5–)6–6.7–7.5(–8) μm; clavate; with 4 sterigmata. Basidioles first cylindrical, then clavate, ca. 3–6 μm wide. Marginal cells (10–)12.5–15.4–18.5(–21.5) × (3.5–)3.7–4.9–6(–8) μm; mostly clavate and similar to basidioles on lamellae sides, but shorter, sometimes fusiform and apically constricted. Pileipellis of mainly one layer of inflated cells; hyphal terminations with brownish parietal pigments, occasionally with thickened walls up to 0.5 μm. Terminal cells near pileus margin (14.5–)20–26.2–32.5(–40) × (7.5–)12–16.1–20.5(–24) μm; sphaeropedunculate, obpyriform or utriform, rarely clavate; subterminal cells often lobate, usually narrower and implemented in intricate hyphae of subpellis. Terminal cells near pileus centre (15–)19–25.2–31.3(–40.5) × (5.5–)9–12.4–16(–21) μm; usually obpyriform, clavate, subglobose or ellipsoid, occasionally sphaeropedunculate. Caulocystidia (10.5–)15.5–19.6–23.5(–27) × (3.5–)4.5–5.8–7(–8) μm; clavate, rarely cylindrical, apically obtuse, ascending or erect, in large, dense clusters; thin-walled, with brownish parietal pigments. Clamp connections present (Figs 7, 8).

#### Notes

*Dermolomahymenocephalum* was originally placed in the genus *Collybia* by Smith (1941) and later hesitantly combined in *Dermoloma* by Singer (1962), a few years after the introduction of the genus *Dermoloma* in Europe (invalidly by Singer (1955), but later validated by Herink (1958)). However, the original description mentioned characters typical for the genus *Dermoloma*, including inflated pedicellate cells in the pileipellis, amyloid spores, context with farinaceous taste (but indistinct odour), fragile context etc. Singer (1975) classified the species in D.sect.Atrobrunnea with some hesitation. Based on his doubts and suggestions, Redhead (1984) combined this species into the genus *Hydropus*. Recently, Sánchez-García et al. (2021) confirmed by the sequencing of the type specimen that this species belongs in *Dermoloma*. *Dermolomahymenocephalum* is clearly different from the four North American species present in the phylogeny of Sánchez-García et al. (2021) and it clustered with four more collections from Smith’s fungarium in the phylogenetic tree. The long branch in the ITS tree is possibly caused by a low sequence quality of the type sample and at least three (possibly all five) collections from Smith’s fungarium may represent *D.hymenocephalum*. It is characterised by shorter spores compared to other North American *Dermoloma* species with amyloid spores.

### Dermoloma inconspicuum

Dennis, 1961

6A947659-D476-5A4B-A264-509D589C8928

Dermoloma inconspicuum Dennis, Kew Bull. 15(1): 78. 1961.

#### Materials

- **Type status:** Holotype. **Location:** country: Venezuela; municipality: Caracas; locality: Dpo. Federal, Botanical Garden; locationRemarks: on bare soil under trees; **Identification:** identifiedBy: R. W. G. Dennis; dateIdentified: 03-07-1958; **Record Level:** collectionID: *Dennis 1131* [K(M)147991]; institutionCode: K (M)

#### Description

**Figure 9.**
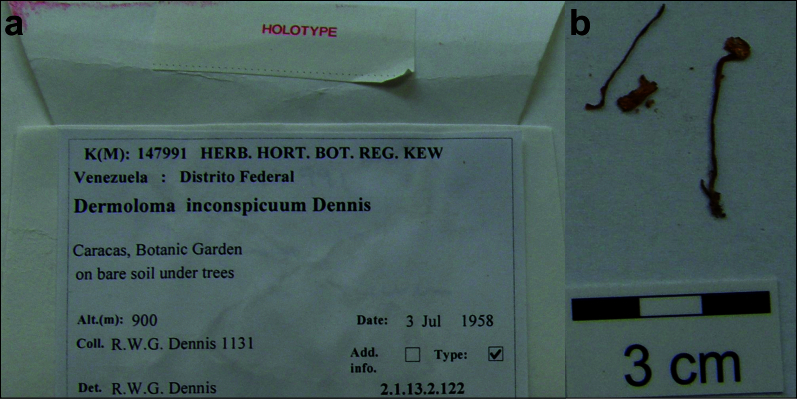
*Dermolomainconspicuum* [K(M)147991, holotype], details of the type specimen. **a** Original label of the type specimen; **b** Basidiomata of the type collection.

**Figure 10.**
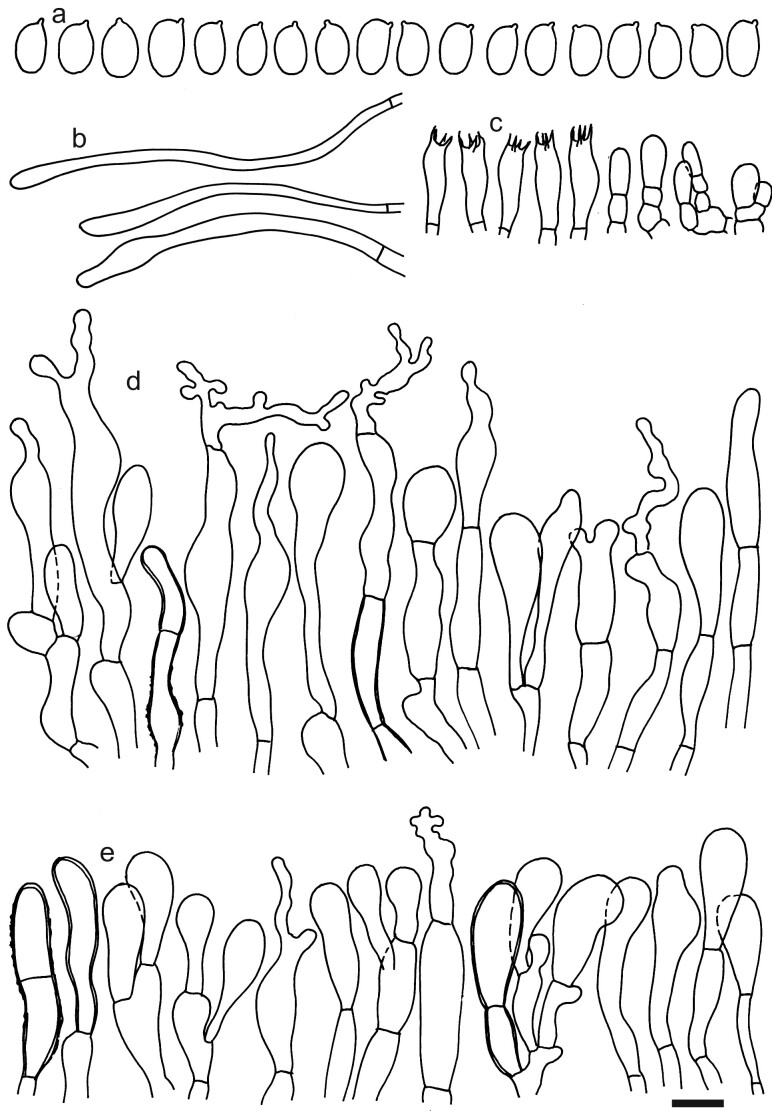
*Dermolomainconspicuum* [K(M)147991, holotype], microscopic elements. Scale bar = 5 µm for spores and 10 µm for the other elements. **a** Spores; **b** Caulocystidia; **c** Basidia and basidioles; **d** Pileipellis elements near the pileus margin; **e** Pileipellis elements near the pileus centre.

Spores (4.9–)5.1–5.3–5.6(–5.7) × (2.9–)3.0–3.2–3.4(–3.5) μm; oblong, Q = (1.54–)1.59–1.67–1.74(–1.84); inamyloid, not dextrinoid, thin-walled, with oil drops or refringent contents in central part; hilar appendage ca. 0.4 μm long. Basidia (13–)15.5–17.0–18.5(–20) × (4.5–)4.8–5.2–5.5(–6) μm; clavate or fusiform; with 4 sterigmata. Basidioles first cylindrical, then clavate, ca. 3.5–7 μm wide. Marginal cells not observed, probably not well differentiated. Pileipellis a trichoderm to almost an hymeniderm, composed of one (or two) layers of mostly clavate cells; hyphal terminations with dark brown to almost black parietal pigments, sometimes also with dark incrusted pigments near basal septa, occasionally with thickened walls up to 0.5 μm; subpellis of relatively short (ca. 10–30 μm) and narrow (ca. 2.5–5 μm) cells. Terminal cells near pileus margin (16–)19.5–34.9–50(–72) × (4–)5.5–7.9–10(–12.5) μm; usually clavate, sometimes fusiform or subcylindrical, apically obtuse or with moniliform, simple, branched to coralloid appendages; subterminal usually shorter, sometimes equally wide and cylindrical or slightly ventricose, sometimes narrower and branched, usually narrower and implemented in intricate hyphae of subpellis. Terminal cells near pileus centre (16–)19.5–28.3–37(–49) × (6–)7.5–9.1–10.7(–15.5) μm; mostly clavate, occasionally sphaeropedunculate, ellipsoid or obpyriform, apically mostly obtuse and rarely with branched appendages. Caulocystidia absent, stipe surface covered by narrow (ca. 1.5–3 μm wide) hyphae with distant septa and very scarce repent hyphal terminations. Clamp connections inconspicuous or absent. (Figs 9, 10).

#### Notes

*Dermolomainconspicuum*, described from Venezuela, was the first member of the genus included in a molecular phylogenetic study and placed close to *Lepiota* (Pers.) Gray in the family *Agaricaceae* Chevall. (Kropp 2008). Previous results of Sánchez-García et al. (2021) confirmed that the majority of studied *Dermoloma* species including the type species belong to the family *Tricholomataceae* and that *D.inconspicuum* is not a member of this genus. The species was placed in the genus *Dermoloma*, based on a hymenidermic pileipellis composed of relatively narrowly clavate, 10–12 μm wide terminal cells (Dennis 1961). The morphology of the type specimen also strongly suggests that this is not a member of the genus *Dermoloma*, because of relatively narrow, on average only 7–9 μm wide terminal cells in the pileipellis with frequent appendages which are often branched.

### Dermoloma intermedium coniferarum

Bon 1986

DA3BAA25-EED2-569F-AC4E-B5C523F28E56

https://ncbi.nlm.nih.gov/nuccore/MW307771

Dermoloma intermedium var. coniferarum Bon, Docums Mycol. 65: 51. 1986.

#### Materials

- **Type status:** Holotype. **Occurrence:** occurrenceID: EAF8B396-F45A-5447-87B8-29E242B9577E; **Location:** country: France; county: Argol; municipality: Lambibi; **Identification:** identifiedBy: J. Mornand; dateIdentified: 28.10.1982; **Record Level:** collectionID: Bon 8116 (LIP); institutionCode: LIP

#### Description

**Figure 11.**
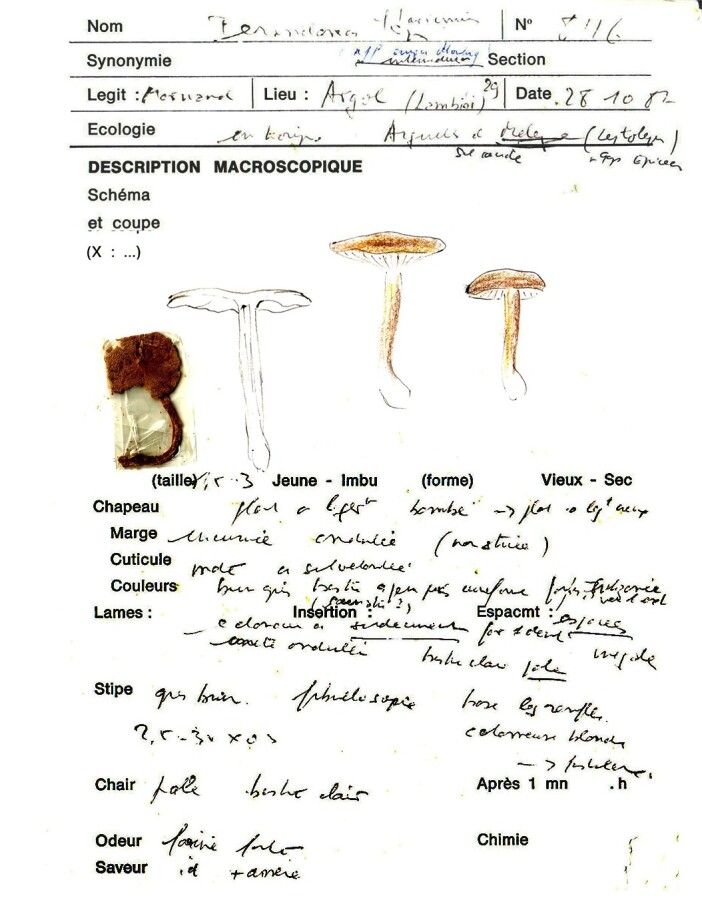
Dermolomaintermediumvar.coniferarum [LIP (Bon 8116), holotype], details of the type specimen, description form.

**Figure 12.**
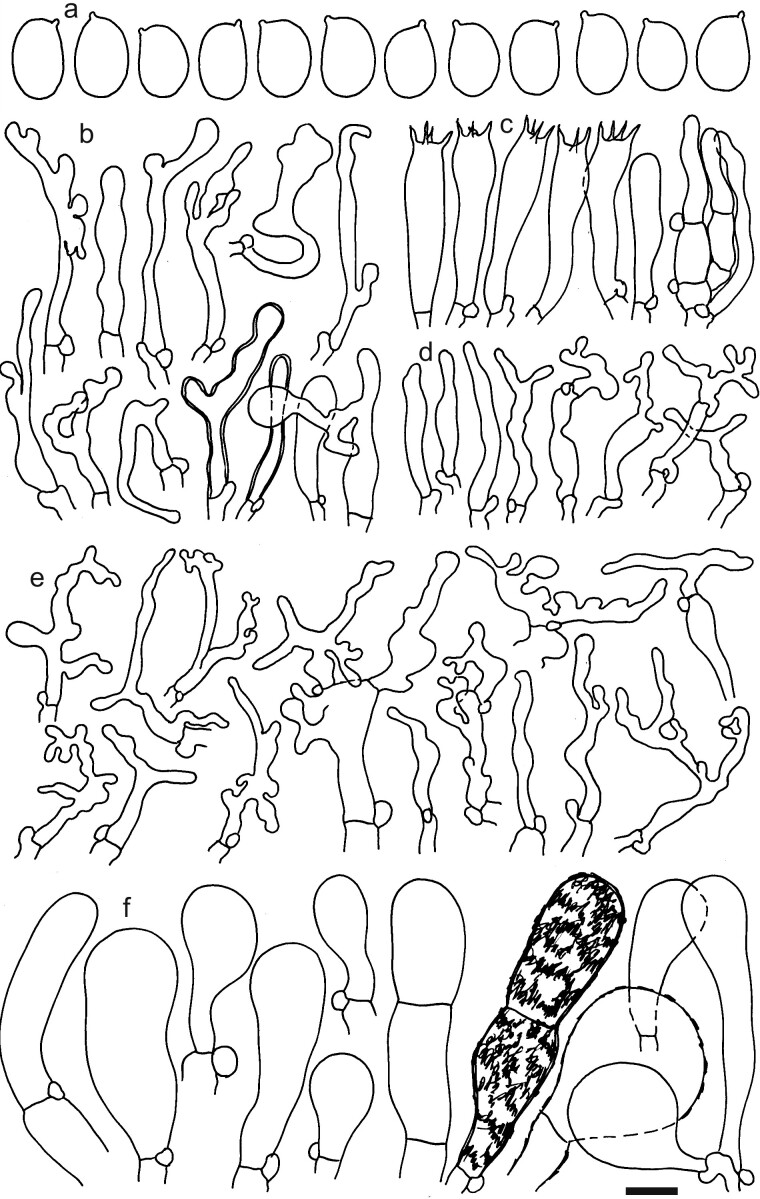
Dermolomaintermediumvar.coniferarum [LIP (Bon 8116), holotype], microscopic elements. Scale bar = 5 µm for spores and 10 µm for the other elements. **a** Spores; **b** Caulocystidia; **c** Basidia and basidioles; **d** Marginal cells; **e** Coralloid hyphae in pileipellis near the pileus margin; **f** Large inflated incrusted hyphal terminations in pileipellis near the pileus margin.

Spores (7–)7.2–7.6–7.9(–8.5) × (4.7–)5.0–5.2–5.5(–5.7) μm; ellipsoid to narrowly ellipsoid, Q = (1.29–)1.39–1.45–1.52(–1.58); amyloid, thin-walled; hilar appendage ca. 0.3–0.8 μm long. Basidia (29–)32–35–38(–40) × (7–)7.5–7.9–8.5(–9) μm; clavate; with 4 sterigmata. Basidioles first cylindrical or lageniform, then clavate, sometimes centrally constricted, ca. 3–7 μm wide. Marginal cells (16–)22.5–34.4–46(–63) × 3–3.9–4.5(–5.5) μm; narrowly fusiform, attenuated or subcylindrical, flexuous, often moniliform, frequently diverticulate or branched (bifurcated or with lateral branches), often nodulose or lobate, sometimes almost coralloid, thin-walled. Pileipellis an intricate trichoderm or a transition to a cutis; terminal cells of two types, large, (22–)27.5–37.5–47.7(–60) × (10–)11.9–16.8–21.5(–30) μm, inflated, clavate or obpyriform cells incrusted by thick yellowish-brownish parietal pigments, mixed with narrower, (24–)28–36.9–46(–58) × (3–)3.5–4.3–5(–6) μm, mainly coralloid, flexuous, lobate, moniliform, not incrusted cells. Caulocystidia (27–)31–40–48.5(–63) × (3.5–)4–5.7–7.5(–9) μm; clavate or subcylindrical, simple, branched or coralloid, lobate, moniliform, occasionally inflated, apically obtuse; thin-walled or occasionally with thickened walls (up to 0.5 μm). Clamp connections present (Figs 11, 12).

#### Notes

Dermolomaintermediumvar.coniferarum was proved not to be a member of the genus *Dermoloma*. Previous sequencing of type material indicated that it is identical with *Pseudolaccariapachyphylla* (Fr.) Vizzini & Contu (Sánchez-García et al. 2021). Our morphological observations of the type specimen revealed the presence of coralloid hyphal terminations in the pileipellis mixed with large, incrusted and inflated elements, which demonstrated that this taxon has a very different pileipellis structure compared to *Dermoloma*. Some authors of this study also misidentified *P.pachyphylla* in the field as a *Dermoloma* species, suggesting that the correct recognition of this species in the field requires some experience. We recognised the bitter taste and the radial rimulose or squamulose pattern on the pileus surface in the mature stage or in dry conditions as a useful diagnostic character of *P.pachyphylla* (see also Lavorato et al. (2015)). The pileus surface of *Dermoloma* species becomes occasionally cracked when exposed to dry and windy conditions, but the cracking is irregular and sometimes concentric.

### Dermoloma longibasidiatum

Contu, Consiglio & Setti, 2008

DE543E66-E023-5193-9022-9A57C4F6AEAF

Dermoloma longibasidiatum Contu, Consiglio & Setti, Micol. Veg. Medit. 22(2): 110. 2008.

#### Materials

- **Type status:** Holotype. **Location:** country: Italy; stateProvince: Trentino-Alto Adige; county: Pergine; municipality: Susà; locationRemarks: grassland on margin of mixed forest of *Fagus* and *Larix*; **Identification:** identifiedBy: G. Consiglio, G. Marasca, B. Oss-Emer; dateIdentified: 30-10-1993; **Record Level:** collectionID: *GC93318* (AMB); institutionCode: AMB

#### Description

**Figure 13.**
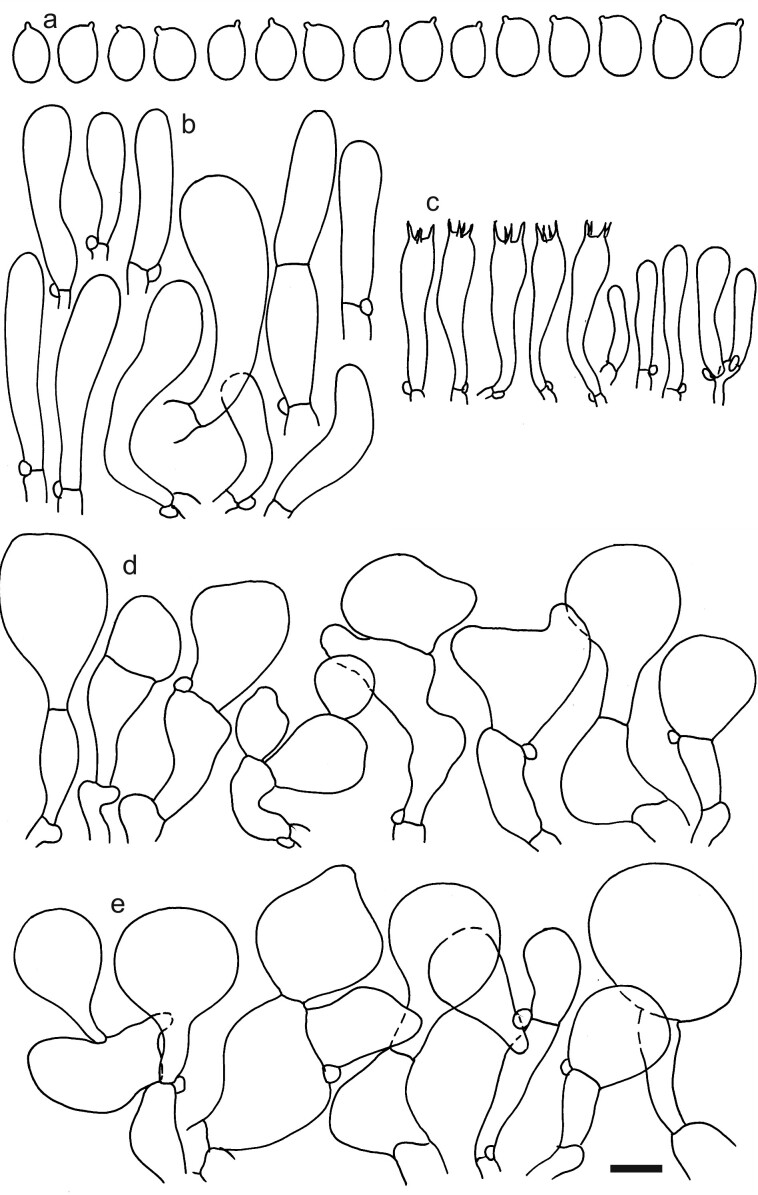
*Dermolomalongibasidiatum* [AMB (GC93318), holotype], microscopic elements. Scale bar = 5 µm for spores and 10 µm for the other elements. **a** Spores; **b** Caulocystidia; **c** Basidia and basidioles; **d** Pileipellis elements near the pileus margin; **e** Pileipellis elements near the pileus centre.

Spores (5.1–)5.3–5.6–5.9(–6.2) × (3.6–)4–4.2–4.5(–4.7) μm; broadly to narrowly ellipsoid, Q = (1.21–)1.26–1.33–1.39(–1.5); inamyloid, not dextrinoid, thin-walled; hilar appendage ca. 0.5 μm long. Basidia (28–)30–32.5–35(–38) × (6.5–)6.7–7.2–7.7(–8) μm; clavate; with 4 sterigmata. Basidioles ellipsoid, cylindrical or clavate, ca. 3.5–6 μm wide. Marginal cells not differentiated. Pileipellis composed of two or three layers of inflated cells; hyphal terminations thin-walled, often with dark incrusted pigments near basal septa and on subterminal cells; subpellis pseudoparenchymatous, of irregularly shaped, 5–28 μm wide elements. Terminal cells near pileus margin (14–)19–26.8–34.5(–53) × (6–)11.5–14.6–18(–22) μm; obpyriform or sphaeropedunculate, apically obtuse; subterminal usually inflated, branched or not, obpyriform or subglobose. Terminal cells near pileus centre (13–)20.5–29.5–38.5(–55) × (8–)11.5–19.2–27(–43) μm; clavate, sphaeropedunculate, obpyriform or subglobose, often lobate, apically mostly obtuse. Caulocystidia (20–)27.5–36.4–45.5(–55) × 7.5–9.6–11.5(–16.5) μm; cylindrical to clavate, apically obtuse; thin-walled, sometimes clustered in tufts. Clamp connections present (Fig. 13).

#### Notes

*Dermolomalongibasidiatum*, described from Italy, was defined morphologically as a species similar to *D.atrocinereum*, but distinguished by longer basidia (33–43 μm long, Contu et al. (2008)). The type sequencing failed, but according to our morphological examination, the length of the basidia falls within the range of *D.atrocinereum* (on average 29–32.5 μm according to our unpublished observations on collections used in the phylogeny of Sánchez-García et al. (2021)) and we, therefore, consider *D.longibasidiatum* to be a later synonym of this species.

### Dermoloma pragense

Kubička, 1975 [described as ‘pragensis’]

41AED2A6-1046-5684-857D-B4320A5C4580

Dermoloma pragense Kubička, Česká Mycol. 29: 31. 1975 [described as ‘pragensis’]

#### Materials

- **Type status:** Holotype. **Location:** country: Czechia; municipality: Prague; locality: Kinského sady; locationRemarks: in grass; **Identification:** identifiedBy: E. Wichanský; dateIdentified: 22-06-1965; **Record Level:** collectionID: PRM 611173; institutionCode: PRM

#### Description

**Figure 14.**
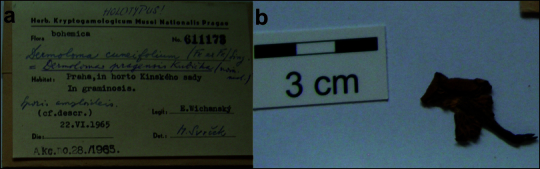
*Dermolomapragense* (PRM 611173, holotype), details of the type specimen. **a** Original label of the type specimen; **b** Basidioma of the type collection.

**Figure 15.**
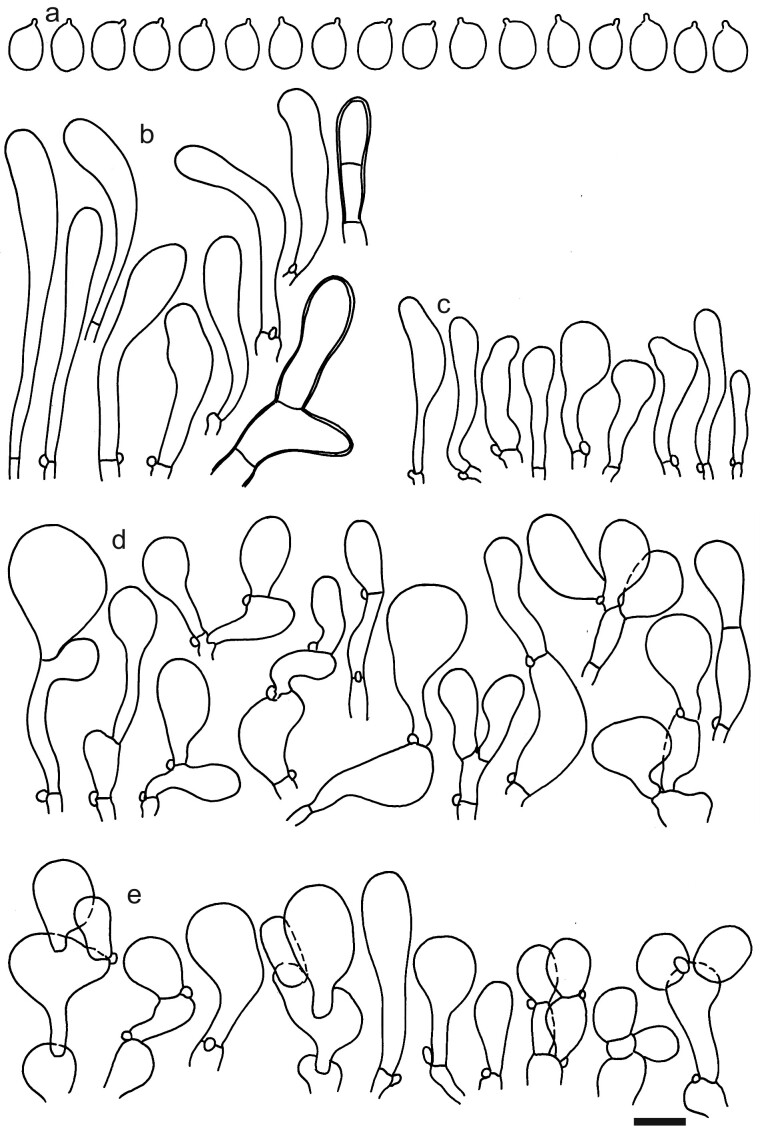
*Dermolomapragense* (PRM 611173, holotype), microscopic elements. Scale bar = 5 µm for spores and 10 µm for the other elements. **a** Spores; **b** Caulocystidia; **c** Marginal cells; **d** Pileipellis elements near the pileus margin; **e** Pileipellis elements near the pileus centre.

Spores 4.4–4.8–5.1(–5.9) × (3.4–)3.6–3.8–4.1(–4.7) μm; broadly ellipsoid, Q = (1.10–)1.17–1.25–1.32(–1.37); amyloid, thin-walled; hilar appendage ca. 0.5–1 μm long. Basidia ca. 17–20 × 5–6 μm; clavate; with 4 sterigmata. Basidioles ellipsoid, cylindrical or clavate, ca. 3–5 μm wide. Marginal cells (16–)22–27.2–32.5(–40) × (3.5–)5–6.5–8(–10.5) μm, clavate, occasionally subcylindrical or subcapitate, often lobate, apically usually obtuse, thin walled. Pileipellis composed of two or three layers of inflated cells; hyphal terminations thin-walled; subpellis pseudoparenchymatous, of irregularly-shaped, 3–8 μm wide elements. Terminal cells near pileus margin 16–26.8–39.5(–70) × (6–)8.5–13.6–18(–26) μm; obpyriform or sphaeropedunculate, apically obtuse; subterminal cells usually inflated, branched or not, obpyriform, ventricose or subcylindrical, sometimes lobate. Terminal cells near pileus centre 9–18.8–28.5(–42) × (7–)7.5–10.3–13(–15) μm; subglobose, ellipsoid, obpyriform or sphaeropedunculate, apically obtuse. Caulocystidia (14–)29–43.1–57.5(–70) × (7–)7.5–8.6–10(–10.5) μm; clavate, rarely ellipsoid, apically obtuse; thin-walled or occasionally with thickened walls (up to 1 μm). Clamp connections present (Figs 14, 15).

#### Notes

*Dermolomapragense*, described from Czechia (former Czechoslovakia), was originally recognised by its amyloid and relatively small spores. There was some nomenclatural confusion about the validity of the name because it was introduced in the key without a detailed description (Kubička 1975). However, the diagnostic characters of the species are described in Latin as “Sporae amyloideae: Sp. 5–6 × 3.5–4.5 μm” and there is a reference to the type specimen (PRM611173), which complies with the requirements for valid publication (Turland et al. 2018, Art. 39.1). Bon (1986) later intended to validate the name at varietal rank as D.pseudocuneifoliumvar.pragense Bon, but because he selected his own collection as the type, he published a new name at the variety rank. Ballero and Contu (1988) combined Bon's variety at species rank and their name is a heterotypic homonym of Kubička's name. Our study is based on the type specimen designated by Kubička (1975) and previously reported by Svrček (1966) as *D.cuneifolium*. The type has very small spores (on average 4.8 × 3.8 μm, Q = 1.25) which agrees only with the spore dimensions of the *Dermoloma* collection SAV F-20229 included in phylogeny of Sánchez-García et al. (2021). However, the holotype collection of *D.pragense* differs in having much larger basidiomata (pileus 30 mm in diameter, stipe 5–6 mm wide) according to Svrček (1966), while SAV F-20229 has pilei 6–8 mm in diameter and stipes up to 1.5 mm wide. In our opinion, *D.pragense* may represent a taxon which is not represented amongst our recent collections.

### Dermoloma pseudocuneifolium

Herink ex Bon, 1986

4973CBF8-D1D9-5B2C-A71F-1A67786D01B6

Dermoloma pseudocuneifolium Herink ex Bon, Doc. Mycol. 17(65): 52. 1986.
Earlier invalid name: *Dermolomapseudocuneifolium* Herink, Acta Musei Horti bot. Bohemiae 1: 62. 1958. [nom. inval., without Latin description]

#### Materials

- **Type status:** Holotype. **Location:** country: France; county: Somme; municipality: Saint-Valery-sur-Somme; **Identification:** identifiedBy: M. Bon; dateIdentified: 10-1968; **Record Level:** collectionID: *Bon 81006* (LIP); institutionCode: LIP

#### Description

**Figure 16.**
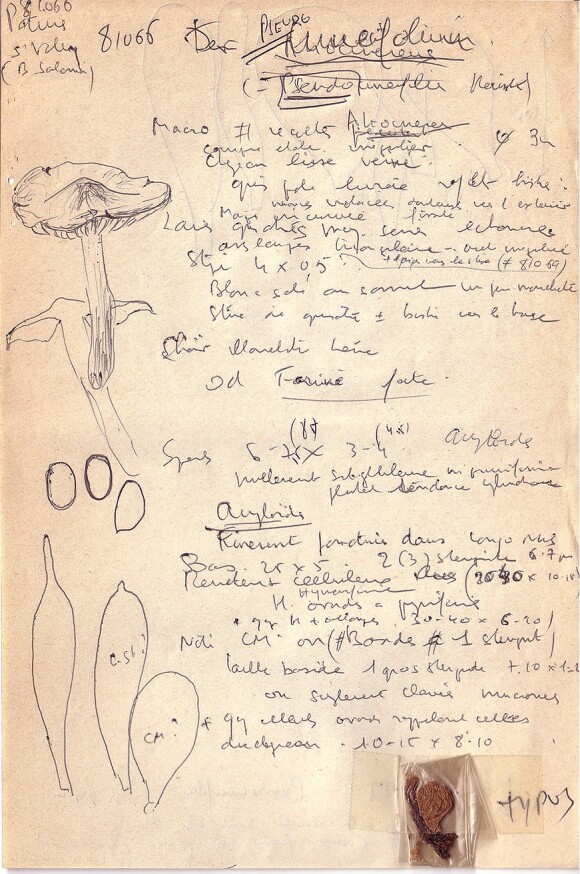
*Dermolomapseudocuneifolium* [LIP (Bon 81006), holotype], details of the type specimen, original field and micromorphological notes.

**Figure 17.**
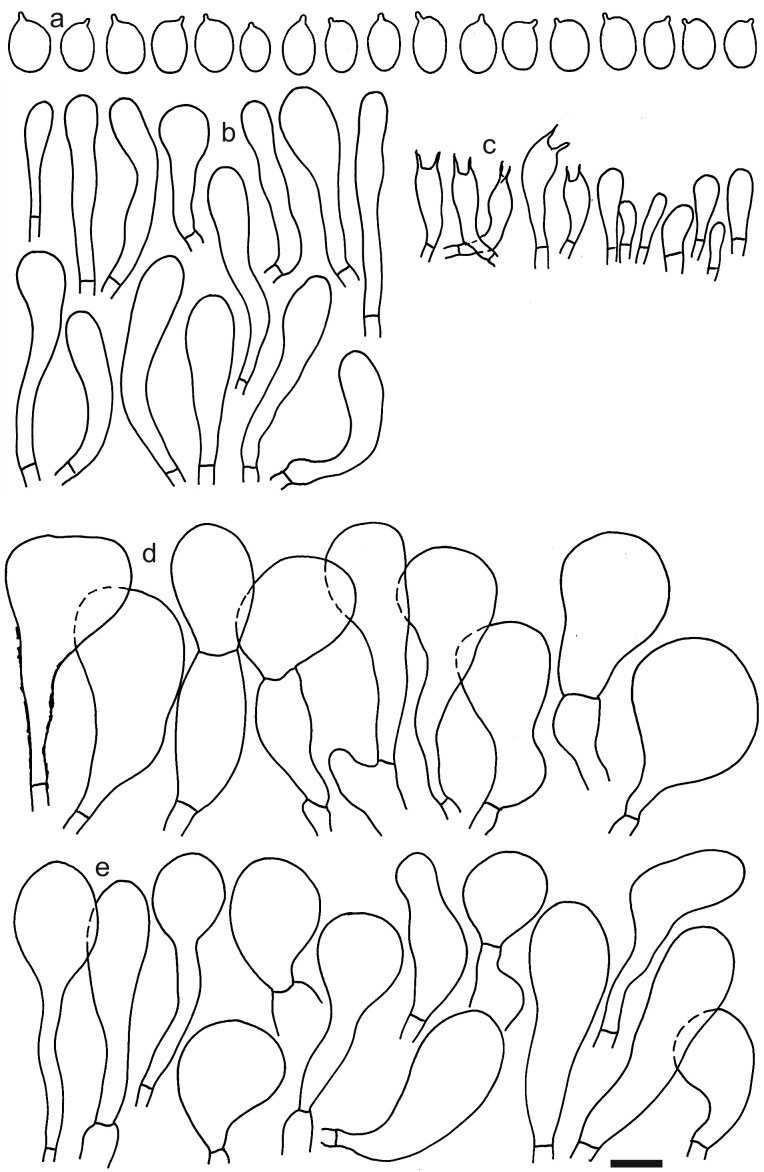
*Dermolomapseudocuneifolium* [LIP (Bon 81006), holotype], microscopic elements. Scale bar = 5 µm for spores and 10 µm for the other elements. **a** Spores; **b** Caulocystidia; **c** Basidia and basidioles; **d** Pileipellis elements near the pileus margin; **e** Pileipellis elements near the pileus centre.

Spores (4.2–)4.8–5.2–5.6(–5.9) × (3–)3.2–3.5–3.9(–4.3) μm; ellipsoid to narrowly ellipsoid, Q = (1.30–)1.37–1.49–1.62(–1.83); inamyloid, not dextrinoid, thin-walled, dispersed also thick-walled and dextrinoid; hilar appendage ca. 0.8–1 μm long. Basidia (14–)16.5–18.2–20(–22) × (4.5–)5–5.7–6.5(–7) μm; clavate; with 2 sterigmata, thin-walled, occasionally also thick-walled and dextrinoid. Basidioles cylindrical or clavate, ca. 2.5–5.5 μm wide. Marginal cells not differentiated. Pileipellis composed of one or two layers of inflated cells; hyphal terminations thin-walled, often with dark incrusted pigments near basal septa and on subterminal cells; subpellis pseudoparenchymatous, of irregularly-shaped, 5–12 μm wide elements. Terminal cells near pileus margin (27–)30–43.8–58(–82) × (15.5–)18–23.6–29(–40) μm; obpyriform or sphaeropedunculate, apically obtuse; subterminal ventricose-inflated or subcylindrical, branched or not. Terminal cells near pileus centre (20–)29–39.3–49.5(–61) × (12–)13–16.2–19(–23) μm; clavate, sphaeropedunculate or obpyriform, apically obtuse. Caulocystidia (25–)33.5–41.0–48(–53) × (5.5–)6.5–8.3–10(–11.5) μm; clavate, apically obtuse; thin-walled. Clamp connections absent (Figs 16, 17).

#### Notes

*Dermolomapseudocuneifolium* was introduced by Herink (1958) as an invalid name (no Latin description) and later validated by Bon (1986) who provided a Latin diagnosis and designated a personal collection as the holotype. Our sequencing of the type was unsuccessful, but the type specimen (a single basidiome) showed bisporic basidia without clamp connections and inamyloid, narrow spores, on average 5.2 × 3.5 μm, Q = 1.49. These spores are very narrow and clearly match those of *D.bellerianum* Bon presented in the phylogeny by Sánchez-García et al. (2021). However, Bon’s concept was based on a misapplication of *D.cuneifolium* by Josserand (1943) and the protologue as well as Bon's notes attached to the type specimen both describe the spores as amyloid, 7.5–9 × 4–5 μm. Such a discrepancy suggests a confusion on Bon’s part, the origin of which could not be traced; inamyloid spores of the type specimen are also contrary to the current name used for a species with amyloid spores (Wilhelm 1992, Arnolds 1993, Arnolds 1995, Contu et al. 2008, Sánchez-García et al. 2021). Therefore, we here consider it a *nomen dubium*.

## Discussion

Amongst the 23 validly published *Dermoloma* names in Europe and North America at species and lower rank, Sánchez-García et al. (2021) were able to obtain ITS sequences from type collections of nine taxa prior to this study. Two of these successfully sequenced types are described here and the morphology of the pileipellis structure was congruent with their classification, strongly supporting the placement of *D.hymenocephalum* as a member of the genus *Dermoloma* and D.intermediumvar.coniferarum as a synonym of *Pseudolaccariapachyphylla*. We also provided morphological evidence for excluding *D.hybridum* and *D.inconspicuum* from *Dermoloma*. The morphology of *D.atrobrunneum*, *D.hymenocephalum* and *D.pragense* suggested that these names correspond to taxa without a recent record. The other three studied types, D.cuneifoliumvar.punctipes, *D.longibasidiatum* and *D.pseudocuneifolium*, are probably synonyms of other previously published *Dermoloma* names, but could not be unambiguously assigned to any of them by morphological observations and original descriptions without DNA data. Molecular analysis of old hebarium types represent challenges in all aspects of molecular workflow. It frequently results in highly fragmented DNA coupled with multiple fungal contaminations and subsequent poor PCR performance with unspecific amplifications (Forin et al. 2018). In order to increase success in ITS amplification from degraded samples, development of highly specific PCR primers is often needed (Bradshaw et al. 2022).

There are six other European *Dermoloma* names whose types were not successfully sequenced: *D.bellerianum*, *D.fuscobrunneum* P.D. Orton, *D.intermedium* Bon, *D.josserandii* P.D. Orton, *D.magicum* Arnolds and *D.murinellum* E. Horak. Based on morphological observations of the type specimens, these names were assigned to phylogenetically defined species (Adamčíková et al. 2025. In order to stabilise these species concepts, epitypes were selected for each of them amongst recently collected and sequenced collections.

Amongst 22 validly published European names, two *Dermoloma* type collections remained inaccessible to us. A loan request for *D.coryleti* Singer & Clémençon to F (Field Museum of Natural History, Chicago, USA) was not successful, but the original diagnosis (Singer and Clémençon 1971) describes relatively large spores, absence of distinct odour and field characters which strongly suggest that this species is not a member of the genus *Dermoloma*. *Dermolomaclavicystis* Voto was described recently (Voto 2022), but since an ITS sequence was made available by the author, the phylogenetic placement of this species can be identified and it does not require further analyses from our part. However, this species also needs urgent morphological revision, because its morphological circumscription is insufficiently brief and is based on the presence of marginal cells (presented by the author as cheilocystidia) which proved to be present and well differenciated in the majority of species within D.subgenusAmylospora. The present study is crucial for an efficient and stable use of the oldest *Dermoloma* names. Explaining concepts of older names only documented by brief and incomplete protologues is a good practice contributing to nomenclatural stability and important for the consolidation of further scientific finds (Yurkov et al. 2021). This study is important for aiding in the delimitation of *Dermoloma*, but sometimes the conclusion about identity of type specimens has limitations due to low quality of the fungal material and absence of distinct morphological differences amongst species (Adamčíková et al. 2025).

## Supplementary Material

XML Treatment for Dermoloma atrobrunneum

XML Treatment for Dermoloma cuneifolium cuneifolium

XML Treatment for Dermoloma hybridum

XML Treatment for Dermoloma hymenocephalum

XML Treatment for Dermoloma inconspicuum

XML Treatment for Dermoloma intermedium coniferarum

XML Treatment for Dermoloma longibasidiatum

XML Treatment for Dermoloma pragense

XML Treatment for Dermoloma pseudocuneifolium

## Figures and Tables

**Table 1.** Studied *Dermoloma* types with information about the fungaria where they are deposited.

Taxon name	Country of the type origin	Fungarium name	Acronym
*D.atrobrunneum*	Trinidad	Kew Royal Botanic Gardens, England, UK	K (M)
D.cuneifoliumvar.punctipes	The Netherlands	Naturalis Biodiversity Center, Leiden, The Netherlands	L
*D.hybridum*	France	Conservatoire et Jardin botaniques de la Ville de Genève, Switzerland	G
*D.hymenocephalum*	USA	University of Michigan, Michigan, USA	MICH
*D.inconspicuum*	Venezuela	Kew Royal Botanic Gardens, England, UK	K (M)
D.intermediumvar.coniferarum	France	Université de Lille, Lille, France	LIP
*D.longibasidiatum*	Italy	Associazione Micologica Bresadola, Trento, Italy	AMB
*D.pragense*	Czechia	National Museum, Prague, Czechia	PRM
*D.pseudocuneifolium*	France	Université de Lille, Lille, France	LIP

## References

[B13306742] Adamčíková K, Kiran M, Caboň M, Matheny BP, Sánchez-García M, Arnolds E, Caboňová M, Corriol G, Dima B, Friebes G, Griffith GW, Grootmyers D, Harries D, Karich A, Mešič A, Mihajlevič M, Moreau PA, Pošta A, Shapkin V, Tkalčec Z, Vizzini A, Vondrovicová L, Adamčík S, Jančovičová S (2025). A phylogenetic and morphological study of the genus *Dermoloma* (Agaricales, Tricholomataceae) in Europe and North America exposes inefficiency of opportunistic species descriptions. IMA Fungus.

[B12960099] Arnolds E (1992). Notulae ad Floram Agaricinam Neerlandicam – XIX. A revision of *Dermoloma* (J. Lange) Sing. – 1.. Persoonia.

[B12960108] Arnolds E (1993). Notulae ad Floram Agaricinam Neerlandicam – XX. A revision of *Dermoloma* (J. Lange) Sing. – 2.. Persoonia.

[B12960117] Arnolds E, Bas C, Kuyper TW, Noordeloos ME, Vellinga EC (1995). Flora Agaricina Neerlandica.

[B12960130] Ballero M, Contu M (1988). Inquadramento delle specie del genere *Dermoloma* (Lange) Singer ex Herink presenti in Europa. Boletim da Sociedade Broteriana.

[B12960139] Bon M (1979). *Dermolomaintermedium* M. Bon 1979 (espèce charnière entre les genres *Dermoloma* et *Hygrotrama* Singer) et la famille des Dermolomataceae (Bon) B.. Bulletin Annuel de la Fédération Centre-Est d'Histoire Naturelle et de Mycologie.

[B12960148] Bon M (1986). Novitates. Validations et taxons nouveaux. Documents Mycologiques.

[B13310443] Bradshaw Michael J., Carey Julie, Liu Miao, Bartholomew Holly P., Jurick Wayne M., Hambleton Sarah, Hendricks Dylan, Schnittler Martin, Scholler Markus (2022). Genetic time traveling: sequencing old herbarium specimens, including the oldest herbarium specimen sequenced from kingdom Fungi, reveals the population structure of an agriculturally significant rust. New Phytologist.

[B12959921] Caboň Miroslav, Galvánek Dobromil, Detheridge Andrew P., Griffith Gareth W., Maráková Silvia, Adamčík Slavomír (2021). Mulching has negative impact on fungal and plant diversity in Slovak oligotrophic grasslands. Basic and Applied Ecology.

[B12960157] Contu M, Consiglio G, Setti L (2008). Studi sul genere *Dermoloma* (Basidiomycota, Tricholomataceae). Micologia e Vegetazione Mediterranea.

[B12959932] Dayarathne M C, Boonmee S, Braun U, Crous P W, Daranagama D A, Dissanayak A J, Ekanayak H, Jayawardena R, Jones E B G, Maharachchikumbura S S N, Perera R H, Phillips A J L, Stadler M, Thambugala K M, Wanasinghe D N, Zhao Q, Hyde K D, Jeewon R (2016). Taxonomic utility of old names in current fungal classification and nomenclature: Conflicts, confusion & clarifications. Mycosphere.

[B12960166] Dennis R W G (1951). Some Agaricaceae of Trinidad and Venezuela. Leucosporae: Part I.. Transaction of the British Mycological Society.

[B12960175] Dennis R W G (1961). Fungi venezuelani: IV. Agaricales. Kew Bulletin.

[B13306941] Forin Niccolò, Nigris Sebastiano, Voyron Samuele, Girlanda Mariangela, Vizzini Alfredo, Casadoro Giorgio, Baldan Barbara (2018). Next generation sequencing of ancient fungal specimens: The case of the Saccardo Mycological Herbarium. Frontiers in Ecology and Evolution.

[B12960186] Herink J (1958). Šťavnatkovité houby pahorku “Velká Horka” u Mnichova Hradiště. Species familiae *Hygrophoracearum*, collem “Velká Horka” dictum prope Mnichovo Hradiště habitantes.. Acta Musei Horti Bot. Bohemiae. Přírodní Vědy.

[B12960195] Josserand M (1943). Notes critiques sur quelques champignons de la région lyonnaise (3e Serié). Bulletin Trimestrial de la Société Mycologique de France.

[B12960003] Koukol Ondřej, Delgado Gregorio (2021). Why morphology matters: the negative consequences of hasty descriptions of putative novelties in asexual ascomycetes. IMA Fungus.

[B12960240] Kropp BR (2008). *Dermolomainconspicuum* from Belize with molecular support for its placement in the *Agaricaceae*. Mycotaxon.

[B12960249] Kubička J (1975). Houby státní prírodní reservace “Vyšenské kopce” u Českého Krumlova. Czech Mycology.

[B12960258] Kühner R (1947). Quelques agarics rares, critiques, ou noveaux de la région de Besancon. Annales Scientifiques de Franche-Comté.

[B12960030] Lavorato Carmine, Vizzini Alfredo, Ge Zai-Wei, Contu Marco (2015). Redescription of *Clitocybeumbrinopurpurascens* (Basidiomycota, Agaricales) and revision of *Neohygrophorus* and *Pseudoomphalina*. Phytotaxa.

[B12960267] Melzer V (1924). L'ornementation des spores de Russules. Bulletin Trimestriel de la Société Mycologique de France.

[B12960278] Redhead S A (1984). Mycological observations, 4-12: on *Kuehneromyces*, *Stropharia*, *Marasmius*, *Mycena*, *Geopetalum*, *Omphalopsis*, *Phaeomarasmius*, *Naucoria* and *Prunulus*. Sydowia.

[B12960048] Sánchez-García Marisol, Adamčíková Katarína, Moreau Pierre-Arthur, Vizzini Alfredo, Jančovičová Soňa, Kiran Munazza, Caboň Miroslav, Matheny P. Brandon, Adamčík Slavomír (2021). The genus *Dermoloma* is more diverse than expected and forms a monophyletic lineage in the Tricholomataceae. Mycological Progress.

[B12960289] Singer R (1955). Type Studies on Basidiomycetes VIII. Sydowia.

[B12960298] Singer R (1962). Type studies on Agarics IV. Sydowia.

[B12960317] Singer R, Clémençon H (1971). Neue Arten von Agaricales. Schweizerische Zeitschrift für Pilzkunde.

[B12960307] Singer R (1975). The Agaricales in modern taxonomy.

[B12960327] Smith A H (1941). New and unusual agarics from Michigan II. Papers of the Michigan Academy of Science, Arts and Letters.

[B12960339] Svrček M (1966). Agaricales aus Böhmen 2. Czech Mycology.

[B12960349] Turland N J, Wiersema J H, Barrie F R, Greuter W, Hawksworth D L, Herendeen P S, Knapp S, Kusber W H, Li D Z, Marhold K, May T W, McNeill J, Monro A M, Prado J, Price M J, Smith G F (2018). International Code of Nomenclature for algae, fungi, and plants (Shenzhen Code) adopted by the Nineteenth International Botanical Congress Shenzhen, China, July 2017.

[B12960373] Vellinga E C, Bas C, Kuyper T W, Noordeloos M E, Vellinga E C (1988). Flora Agaricina Neerlandica 1..

[B12960039] Vizzini Alfredo, Consiglio Giovanni, Setti Ledo (2020). Testing spore amyloidity in Agaricales under light microscope: the case study of *Tricholoma*. IMA Fungus.

[B12960394] Voto P (2022). *Dermolomaclavicystis* sp. nov. from the Mediterranean region. Mycological Observations.

[B12960411] Wilhelm M (1992). Drei *Dermoloma*-Arten näher betrachtet: *D.atrocinereum* (Pers. ex Pers.) Herink, *D.cuneifolium* (Fr.) P. D. Orton, und *D.pseudocuneifolium* Herink. Zeitschrift für Mykologie.

[B12960420] Yurkov Andrey, Alves Artur, Bai Feng Yan, Boundy-Mills Kyria, Buzzini Pietro, Čadež Neža, Cardinali Gianluigi, Casaregola Serge, Chaturvedi Vishnu, Collin Valérie, Fell Jack W., Girard Victoria, Groenewald Marizeth, Hagen Ferry, Hittinger Chris Todd, Kachalkin Aleksey V., Kostrzewa Markus, Kouvelis Vassili, Libkind Diego, Liu Xinzhan, Maier Thomas, Meyer Wieland, Péter Gábor, Piątek Marcin, Robert Vincent, Rosa Carlos A., Sampaio Jose Paulo, Sipiczki Matthias, Stadler Marc, Sugita Takashi, Sugiyama Junta, Takagi Hiroshi, Takashima Masako, Turchetti Benedetta, Wang Qi-Ming, Boekhout Teun (2021). Nomenclatural issues concerning cultured yeasts and other fungi: why it is important to avoid unneeded name changes. IMA Fungus.

